# Predicting complications after hepatectomy for hepatocellular carcinoma: a narrative review focused on liver failure

**DOI:** 10.3389/fonc.2026.1952252

**Published:** 2026-10-26

**Authors:** Hongtao Zhang, Jianwang Cai, Zhaoming Yang, Tianhua Ye, Zhijian Zhao, Xun Chen, Jian Wan

**Affiliations:** 1 Jishou University Zhuzhou Clinical College, Medical College, Jishou University, Zhuzhou, Hunan, China; 2 Second Department of Hepatobiliary Pancreatic and Splenic Surgery, Medical Center of Digestive Disease, Zhuzhou Hospital Affiliated to Xiangya School of Medicine, Central South University, Zhuzhou, Hunan, China

**Keywords:** hepatectomy, hepatocellular carcinoma, machine learning, post-hepatectomy liver failure, postoperative complications, prediction model

## Abstract

Hepatectomy is a potentially curative treatment for selected patients with hepatocellular carcinoma (HCC), but postoperative complications remain an important source of morbidity and mortality. This narrative review focuses on prediction models for post-hepatectomy liver failure (PHLF), with additional discussion of selected non-PHLF complications. It emphasizes validation design, transportability, calibration, clinical actionability, and the distinction between evidence from HCC cohorts and cohorts with mixed diagnoses. PHLF has the most mature prediction literature because its risk is closely related to measurable liver reserve, portal hypertension, future liver remnant volume and function, and resection extent. Clinical and serum scores are accessible but generally provide moderate discrimination. Volumetric, functional, and imaging assessments add complementary information, whereas machine learning and radiomics models can capture nonlinear and high-dimensional patterns but often rely on small cohorts, random internal splits, and incomplete calibration reporting. Evidence for bile leakage includes validated clinical scores and a large externally validated machine learning model. Models for postoperative ascites, intra-abdominal infection or abscess, and surgical site infection remain sparse and are often perioperative rather than purely preoperative. Across endpoints, high apparent discrimination has not established clinical benefit. Future studies should prioritize standardized endpoints, adequate effective sample size, independent geographic external validation, calibration, net benefit assessment, and risk updating at successive stages. Current models should be used as decision support tools rather than substitutes for multidisciplinary clinical judgment.

## 1 Introduction

Primary liver cancer is the sixth most commonly diagnosed cancer and the third leading cause of cancer death worldwide, and contemporary Barcelona Clinic Liver Cancer (BCLC) guidance recognizes hepatectomy as a potentially curative treatment for appropriately selected patients with resectable hepatocellular carcinoma (HCC) (1, 2). Clinically relevant post-hepatectomy liver failure (PHLF) remains a major cause of perioperative death after hepatectomy (3, 4). Risk prediction is therefore central to shared decision-making, patient selection, operative planning, and targeted perioperative management.

Prediction-model research after hepatectomy has focused predominantly on PHLF because of its close association with perioperative mortality, measurable determinants of hepatic reserve, and direct relevance to decisions about resection extent and future liver remnant augmentation (5). By contrast, dedicated models for bile leakage, postoperative ascites, intra-abdominal abscess, and surgical site infection are fewer and methodologically less mature. Their prediction is complicated by stronger dependence on operative technique, perioperative management, and downstream postoperative events, as well as by the limited availability of large multicenter HCC cohorts.

This review goes beyond cataloging model performance. It organizes PHLF prediction by the clinical information used—clinical and serum measures, imaging and functional assessment, and machine learning—while placing models for selected non-PHLF complications in the same evaluative framework. We distinguish studies confined to HCC from those including mixed diagnoses. We also separate random internal splitting, temporal validation at the same center, and independent geographic external validation, and examine whether a prediction can support an action when its predictors become available. This perspective based on endpoint and prediction stage is intended to clarify which tools are closest to clinical use and why dynamic perioperative updating is a priority for future research.

## 2 Post-hepatectomy liver failure risk prediction models

### 2.1 Definition, grading, and clinical rationale for prediction

PHLF is a major cause of perioperative mortality after hepatectomy, although its reported contribution varies with the population and extent of resection (6). The definition has evolved to enable more reproducible comparison across studies. The 2005 “50–50 criteria” (prothrombin time <50% and serum bilirubin >50 μmol/L on postoperative day 5) identified a group with 59% in-hospital mortality (7), and Mullen et al. subsequently proposed peak serum bilirubin >7 mg/dL as a simple criterion for postoperative hepatic insufficiency (8). The 2011 International Study Group of Liver Surgery (ISGLS) consensus defines PHLF as an increased international normalized ratio (INR) and concomitant hyperbilirubinemia according to local laboratory upper limits on or after postoperative day 5. Severity is graded by its effect on clinical management: Grade A requires no change in clinical management, Grade B requires a deviation from routine management without invasive treatment, and Grade C requires invasive treatment. Grades B and C are commonly grouped as clinically relevant PHLF (3).

The reported incidence of clinically relevant PHLF varies with case mix, background liver disease, and resection extent. In addition to its association with mortality, PHLF can prolong hospitalization and increase resource use (6). Its reproducible endpoint, measurable preoperative determinants, and potential to influence operative planning have made PHLF particularly amenable to prediction-model development.

### 2.2 Clinical and serum scoring systems

Clinical and serum scores remain the foundation of PHLF risk assessment because they are inexpensive, reproducible, and widely available. They range from established liver function classifications to objective biochemical scores and combined fibrosis or inflammation indices. Their principal limitation is that they summarize selected aspects of baseline liver disease without directly measuring the volume, regional function, or mechanical properties of the future liver remnant.

The Child-Pugh score, incorporating bilirubin, albumin, prothrombin time/INR, ascites, and encephalopathy, is the oldest widely used liver function assessment; its modified form was introduced for risk stratification in patients with cirrhosis undergoing surgery for variceal bleeding (9). It was not developed specifically for post-hepatectomy risk. Its strengths are simplicity and extensive clinical familiarity, but it contains subjective components and converts continuous variables into coarse categories, limiting granularity among Child-Pugh A resection candidates. The Model for End-Stage Liver Disease (MELD) score was developed to predict survival in advanced liver disease and has also shown limited discrimination for PHLF in surgical cohorts (10, 11).

The 2015 Albumin-Bilirubin (ALBI) grade represented an important advance because it was developed in patients with HCC using only albumin and bilirubin and contains no subjective components (12). ALBI has shown better discrimination than Child-Pugh in some post-hepatectomy cohorts and further separates risk within Child-Pugh A patients (13). Indocyanine green retention at 15 minutes (ICG-R15), a dynamic liver function test incorporated into the classic Makuuchi criteria for selecting resection extent (14), has modest standalone discrimination. The Albumin-Indocyanine Green Evaluation (ALICE) grade combines albumin and ICG-R15 (15), and subsequent work showed that adding portal hypertension improved identification of patients at risk of PHLF (16).

Combined serum scoring systems incorporating fibrosis markers and/or inflammatory and nutritional parameters deliver incremental predictive performance over single biomarkers. For instance, the integrated albumin-bilirubin–fibrosis-4 (ALBI-FIB-4) score yielded an area under the receiver operating characteristic curve (AUC) of 0.783 for PHLF prediction (17), while composite models constructed solely from inflammatory and nutritional indicators demonstrated relatively modest discriminative power (18). Notably, both FIB-4 and the aspartate aminotransferase-to-platelet ratio index (APRI) were initially designed as noninvasive liver fibrosis staging tools (19, 20); they only serve as surrogates of chronic baseline hepatic burden and cannot directly quantify perioperative functional reserve after resection. Taken together, standalone serum indices generally exhibit only moderate ability to identify patients at risk of PHLF.

### 2.3 Imaging, volumetric, functional, and integrated multivariable assessment

Imaging, volumetric, and functional assessments extend PHLF prediction beyond circulating biomarkers by characterizing the quantity and quality of the future liver remnant. Because these measures are frequently combined with clinical and laboratory predictors in conventional multivariable or penalized models, this section also considers integrated models that incorporate these information domains. Model architecture alone is not treated as a separate clinical category.

#### 2.3.1 Volumetric and functional assessment

Anatomical remnant volume does not necessarily represent regional functional reserve. Standardized future liver remnant (sFLR) expresses computed tomography (CT)-derived remnant volume relative to an individualized estimate of total liver volume (21), but this anatomical metric cannot capture segmental functional differences. By contrast, radionuclide methods such as 99mTc-galactosyl human serum albumin clearance can assess regional remnant function and identify intrahepatic functional heterogeneity (22). Among magnetic resonance imaging (MRI)-based assessments, a model combining the Functional Liver Imaging Score (FLIS), ALBI grade, and major resection predicted Grade B/C PHLF with AUCs of 0.746 in development and 0.717 in random internal split validation and outperformed ALBI, MELD, and ICG-R15 in the reported comparisons (23). Two-dimensional shear-wave elastography (2D-SWE) provides a complementary measure of liver stiffness. In the development cohort, liver stiffness achieved an AUC of 0.795, higher than those of ICG-R15 and ALBI. The cutoff of 10.35 kPa yielded a specificity of 76.3% in the internal validation cohort (24). Clinically significant portal hypertension (CSPH), conventionally defined by a hepatic venous pressure gradient of at least 10 mmHg (25), is also relevant to post-hepatectomy risk. In one HCC cohort, substituting CT-based CSPH assessment into existing frameworks modestly increased the AUC of the Wang model from 0.830 to 0.854 and that of the European Association for the Study of the Liver (EASL) framework from 0.694 to 0.724 (26).

Large conventional multivariable models provide background on current PHLF prediction, although comparisons across studies cannot establish the added value of imaging and functional measures. In an international cohort of 2,192 major hepatectomies from 12 centers, a least absolute shrinkage and selection operator (LASSO) model achieved an AUC of 0.780 with preoperative variables and 0.800 after intraoperative variables were added. The pooled cohort was randomly split, so this was an internal validation rather than a center-based external validation (27). A separate National Surgical Quality Improvement Program (NSQIP) nomogram, developed from 10,808 hepatectomies, reported a C-statistic of 0.780 in both the development cohort and a later temporal cohort from the same database. Its population with mixed diagnoses may limit direct application to HCC (28).

Several models have focused specifically on HCC. The APRI-sFLR score combined a fibrosis marker with standardized remnant volume and achieved an AUC of 0.816 for Grade B/C PHLF (29). A preoperative nomogram in 1,172 patients achieved AUCs of 0.818 in development and 0.812 in independent geographic validation (30). Another perioperative nomogram combined laboratory and operative variables and reported a C-index of 0.773 (31). Multicenter investigators have also used ordinal models to estimate the probability of each grade of symptomatic liver failure rather than treating PHLF as a binary outcome (32). Across these larger conventional studies, discrimination was generally 0.780–0.820. Direct comparisons remain difficult because case mix, PHLF definition, timing of predictors, and validation design differed. A systematic review likewise reported a median AUC of 0.790, with values ranging from 0.650 to 0.933 (33).

#### 2.3.2 Hepatobiliary MRI biomarkers and regional functional reserve

Hepatobiliary contrast-enhanced MRI adds information on liver tissue and function to anatomical assessment. Gadoxetic acid (Gd-EOB-DTPA) and gadobenate dimeglumine (Gd-BOPTA) produce enhancement in the hepatobiliary phase through hepatocyte uptake and biliary excretion. Depending on how it is measured, this enhancement may describe the sampled parenchyma, the planned future liver remnant (FLR), or the biliary system. A systematic review of 15 gadoxetic acid-enhanced MRI studies found that these measures were associated with PHLF, but protocols, indices, populations, and outcome definitions varied. Only one study was prospective, and two used separate development and validation cohorts (34).

Chuang et al. combined remnant volume and parenchymal enhancement. The AUC was 0.78 for the remnant liver contrast-enhancement ratio (rCER) and 0.75 for standardized remnant volume, without a significant difference. Because remnant volume was derived by subtracting resected specimen weight from total liver volume, rCER was not evaluated as a fully preoperative measure (35). Other studies focused more directly on the planned FLR. In a prospective portal-vein embolization (PVE) cohort of 36 patients, change in left-lobe enhancement predicted any-grade PHLF with an AUC of 0.854, although only one patient had HCC (36). Among 120 patients with an ICG clearance rate of the remnant (ICG-Krem) below 0.10, the remnant hepatocyte uptake index (rHUI) achieved an AUC of 0.814, compared with 0.725 for ICG-Krem (37). In 333 major hepatectomies, a volume-adjusted liver-to-spleen signal intensity ratio increasing rate in the remnant (vaLSRi-rem) below 0.147 was associated with Grade B/C PHLF (adjusted OR 9.12, 95% CI 2.71–38.00). The OR point estimate was larger in the PVE subgroup, although the confidence interval was wide (OR 21.04, 95% CI 3.61–403.35) (38).

Whole-liver T1 mapping captures the distribution of relaxation times across the liver rather than directly measuring function in the planned FLR. In an exploratory study of 37 minor hepatectomies, the standard deviation of T1 in the hepatobiliary phase yielded an AUC of 0.785, and the 95th percentile remained independently associated with PHLF. ALBI did not show significant discrimination, although the T1-derived AUCs were not significantly different from that of ICG-R15 (39). The exploratory design and small cohort with mixed diagnoses support confirmation in larger validation studies.

The two Gd-BOPTA studies examined parenchymal enhancement and biliary excretion. In 307 patients with HCC from three centers, the liver-to-portal-vein contrast ratio (LPC) achieved an AUC of 0.882, compared with 0.782 for the liver-to-spleen contrast ratio (LSC) and 0.687–0.747 for Child-Pugh, MELD, ALBI, and standardized FLR (40). In a separate cohort of 221 patients with HCC from two centers, the relative enhancement ratio of the biliary system (REB), measured at 90 minutes, achieved an AUC of 0.87, compared with 0.60 for the liver-to-muscle ratio (41). These results suggest that Gd-BOPTA-enhanced MRI may complement conventional liver function assessment. Both studies drew on multicenter cohorts, and evaluation in additional populations and acquisition settings would help establish the wider applicability of the proposed thresholds.

#### 2.3.3 Incremental value of functional imaging and hepatobiliary MRI biomarkers

The incremental value of imaging is most clearly assessed by comparing a clinical model with and without imaging variables. One study included 276 patients with HCC, 98.6% of whom were Child-Pugh A. A model combining radiomics from the hepatobiliary phase, ALBI, and ICG-R15 achieved an AUC of 0.82 in the internal test cohort, compared with 0.71 for the clinical model and 0.79 for radiomics alone. The difference was significant between the combined and clinical models, but not between the combined and radiomics models; calibration and decision-curve analyses were also reported (42). These findings suggest a potential contribution from imaging in this predominantly Child-Pugh A cohort, without establishing a greater benefit in this subgroup. The radiomic features were extracted from the whole non-tumorous liver and do not directly measure FLR function. Because the clinical variables were reselected for the combined model and validation was internal, external studies using a prespecified clinical model are still needed.

Most other studies, including the rHUI analysis (37) and the LPC and REB studies (40, 41), compared an imaging index with existing tests or scores rather than testing the change in performance after imaging was added to a clinical model. These comparisons support a complementary role for imaging, although they provide indirect rather than formal evidence of incremental gain.

For measurements that specifically assess the planned FLR, discordance between acceptable global liver function and poor regional function could prompt reassessment of the planned resection. The broader implications for clinical management are discussed in Section 4.6. Whether such an imaging-guided approach reduces PHLF remains to be tested prospectively.

### 2.4 Machine learning and explainable artificial intelligence

Conventional regression models can incorporate nonlinear terms and interactions, although these usually need to be specified during model development. Machine learning (ML) methods can learn more complex relationships among clinical, biochemical, operative, and imaging variables from the data. This flexibility is particularly appealing for PHLF prediction, where risk reflects the interplay between baseline hepatic reserve, portal hypertension, future liver remnant volume, and the physiological burden of resection. That said, improved apparent discrimination does not always translate into better generalizability or clinical utility—especially when complex algorithms are developed on relatively small datasets.

#### 2.4.1 Machine learning models based on routine clinical variables

Gradient boosting trees — including XGBoost and LightGBM — are frequently used for tabular clinical PHLF data, although their flexibility still requires control of model complexity and careful validation. Tang et al. took a comparative approach, benchmarking 12 different ML algorithms in a cohort of 312 patients with HCC. Their best-performing XGBoost model reached an AUC of 0.983 in the development cohort and 0.981 in a randomly selected 30% internal validation subset, with an AUC of 0.942 in a prospective temporal validation cohort of 62 patients at the same center (43). SHapley Additive exPlanations (SHAP) analysis identified total bilirubin, MELD score, and ICG-R15 as the most influential predictors. Despite these promising results, the small validation cohort from one center leaves substantial uncertainty about geographic transportability, and the unusually high discrimination warrants independent replication.

For larger multi-institutional datasets, Wang et al. developed a Light Gradient Boosting Machine (LightGBM) model across 875 patients from five centers, reporting AUCs of 0.944, 0.870, and 0.822 in the development, internal validation, and test cohorts, respectively (44). SHAP analysis highlighted platelet count, age, serum creatinine, INR, and alpha-fetoprotein as key predictors. Because patients from the participating centers were pooled and randomly split, rather than reserving entire centers for validation, the test set should be viewed as internal validation rather than true geographically independent external validation.

Artificial neural network architectures have also been explored for clinical variable-based prediction. Mai et al. developed an artificial neural network (ANN) incorporating platelet count, prothrombin time, total bilirubin, aspartate aminotransferase, and standardized future liver remnant in 353 HCC patients undergoing hemihepatectomy. The model achieved AUCs of 0.880 in the development cohort and 0.876 in a random internal split validation cohort, outperforming logistic regression and conventional liver function scores within the same dataset (45).

Across tabular ML studies, evidence of a consistent performance advantage over conventional regression remains limited. Direct head-to-head comparison remains difficult because predictor sets, patient populations, and endpoint definitions differ substantially between cohorts. Notably, variable-importance analyses across studies largely emphasize familiar clinical predictors—including bilirubin, ALBI grade, portal hypertension, and resection extent—although feature importance alone does not establish which nonlinear relationships or interactions account for any performance gain.

#### 2.4.2 Imaging-based machine learning

Moving beyond purely clinical variables, imaging-based machine learning offers a route to extract high-dimensional predictive information directly from routine preoperative scans, characterizing image heterogeneity that may be associated with hepatic reserve but is not a direct measure of regional function.

A convolutional neural network trained on multiphase CT achieved an AUC of 0.793 in 265 hemihepatectomy patients, without relying on any additional clinical covariates (46). This study demonstrated the feasibility of extracting predictive risk information directly from routine imaging. Zhong et al. subsequently combined multipatch radiomic features derived from 2D-SWE with clinical variables. Their clinical–radiomics model achieved an AUC of 0.867 during five-fold cross-validation and 0.822 in a temporal validation cohort at the same center, compared with 0.684 for the clinical model alone (47). These findings suggest that imaging features may add predictive information to clinical measures in the studied cohort. Unsupervised radiomics approaches using gadoxetic acid-enhanced MRI have also been applied to identify distinct functional liver phenotypes linked to postoperative risk, offering a data-driven way to stratify parenchymal function beyond subjective visual assessment (48).

Nevertheless, most imaging-based ML studies share key limitations: data acquisition at a single center, modest event numbers, manual or semiautomatic segmentation requiring substantial effort, and sensitivity to scanner settings, reconstruction protocols, and image processing pipelines. These factors limit generalizability and hinder translation to routine clinical practice.

#### 2.4.3 Explainability and methodological limitations

The inherent opacity of complex ML models remains a major barrier to clinical adoption. Clinicians are reluctant to rely on “black box” predictions whose reasoning cannot be understood or verified. SHAP is a widely used framework to address this transparency gap (49). SHAP values quantify the contribution of each predictor to individual model outputs, supporting both population-level feature-importance rankings and patient-specific explanations of why a given individual received a particular risk estimate. Several recent PHLF prediction models have incorporated SHAP analyses, which improve *post-hoc* interpretability.

Importantly, explainability should not be equated with causality or clinical validity. SHAP values describe how variables influence predictions within a fitted model; they do not establish that modifying those variables will alter the risk of PHLF. Explanations may also be unstable in the presence of correlated predictors — for example, bilirubin appearing both as an individual variable and within composite scores such as ALBI or MELD. Furthermore, improved transparency alone does not guarantee that a model will improve patient outcomes or be cost-effective in routine use.

Small PHLF event counts are a particular concern when ML models examine many candidate predictors or compare numerous algorithms and tuning settings. Such analyses can fit chance patterns and overestimate performance. Regularization and limits on model complexity can reduce this risk. Internal validation should repeat all data-dependent steps within the training folds, including imputation, scaling, feature selection, and any oversampling. Hyperparameter tuning should also remain separate from the data used for performance evaluation; using the full dataset for these steps can introduce data leakage (50, 51).

Sample-size planning should consider the total number of patients, PHLF frequency, and complexity of the proposed model. For regression models, Riley et al. relate sample size to candidate predictor parameters, anticipated model fit, and acceptable overfitting, rather than a fixed events-per-variable rule (52). These calculations do not provide a universal threshold for flexible ML algorithms. Development and evaluation sample sizes therefore need separate justification for the chosen approach, with enough PHLF events to estimate performance reliably (50, 51).

In HCC cohorts, differences in liver disease etiology, cirrhosis severity, resection extent, and PHLF definitions may affect performance across centers. Multicenter development should therefore be complemented by evaluation in centers excluded from model development and tuning; random splitting of pooled data does not test performance in a new setting (50, 51).

Reproducibility requires clear reporting of preprocessing, feature extraction, missing-data handling, model selection, and tuning, together with software versions and access to analysis code and the fitted model where feasible. The Transparent Reporting of a multivariable prediction model for Individual Prognosis Or Diagnosis for Artificial Intelligence (TRIPOD+AI) and Prediction model Risk Of Bias ASsessment Tool for Artificial Intelligence (PROBAST+AI) provide complementary guidance for reporting and methodological appraisal (50, 51).

Prospective implementation and impact studies are ultimately required to determine whether risk stratification assisted by machine learning actually changes operative planning, reduces perioperative complications, and improves patient outcomes. At present, these models should be regarded as promising decision support tools rather than established replacements for validated clinical, functional, and volumetric assessment.

### 2.5 Current limitations in PHLF prediction

Several important limitations persist across the PHLF prediction literature. Most studies are retrospective, and many multicenter datasets are pooled before random train-test splitting rather than evaluated in a geographically independent cohort, which may produce optimistic performance estimates. Transportability to populations with different etiologic profiles—such as Western cohorts with alcohol- or metabolic dysfunction-associated liver disease—remains uncertain. We found no prospective implementation trial demonstrating that use of a PHLF prediction model reduces PHLF, mortality, or other patient-centered outcomes, and routine workflow integration remains uncommon. Representative PHLF prediction models are summarized in **Table 1**.

**Table 1:** Representative prediction models and imaging biomarkers for post-hepatectomy liver failure.

Study	Population and endpoint	Approach and principal inputs	Time of prediction	Intended clinical action	Validation classification	Reported performance
Wang et al., 2023 (27)	2,192 major hepatectomies; mixed diagnoses; 12 international centers; Grade B/C PHLF 8.4%	LASSO logistic models using preoperative variables alone or with intraoperative variables	Preoperative model: before surgery; model with intraoperative variables: end of surgery	Potential use: preoperative model—counseling and operative/FLR planning; updated model—postoperative disposition and monitoring	Random internal split (pooled 70:30 split)	AUC 0.780 (preoperative); 0.800 (preoperative + intraoperative)
Dhir et al., 2021 (28)	10,808 NSQIP hepatectomies; mixed diagnoses; Grade B/C PHLF 2.9%	Preoperative nomogram: sex, biliary stent, neoadjuvant therapy, viral hepatitis, concurrent resection, biliary reconstruction, sodium, albumin	Preoperative (procedure variables entered as planned)	Potential use: counseling, patient selection and operative planning	Temporal validation in the same database in the 2017 NSQIP cohort (n=4,367)	C-statistic 0.780 (development); 0.780 (temporal validation in the same database)
Mai et al., 2021 (29)	637 HCC patients; Grade B/C PHLF 15.9%	APRI-sFLR score; prealbumin, cirrhosis, APRI, sFLR and major resection	Preoperative (planned sFLR and resection extent)	Potential use: planned resection and FLR assessment	Development cohort only (no independent validation)	AUC 0.816
Lin et al., 2023 (30)	1,172 HCC patients; development n=977 (PHLF 24.0%); geographic external cohort n=195 (PHLF 16.4%); any-grade ISGLS PHLF	Seven-variable preoperative nomogram including ICG-R15, CSPH and fibrosis/liver reserve indices	Preoperative	Potential use: counseling and further liver reserve assessment	Bootstrap internal validation plus independent geographic external validation	AUC 0.818 (development); 0.812 (independent geographic external validation)
Zhang et al., 2023 (31)	1,081 HBsAg-positive HCC patients; any-grade ISGLS PHLF 10.5%	Nomogram using BMI, ICG-R15, INR, tumor size, inflow occlusion time, operative method and EHBF	End of surgery (requires actual inflow-occlusion time and operative method)	Potential use: immediate postoperative disposition and monitoring	Bootstrap internal validation (no independent validation)	C-index 0.773
Li J et al., 2025 (18)	760 HCC patients; single center	Inflammatory/nutritional nomogram: AAPR, ALBI, GAR, LMR, PNI, INR, APTT and thrombin time	Preoperative	Potential use: baseline risk discussion and perioperative planning	Random internal split (75:25)	C-index 0.691 (development); 0.680 (internal validation)
Zheng et al., 2025 (23)	885 HCC patients; Grade B/C PHLF 7.7%/7.2% in the two cohorts	Gadoxetic acid-enhanced MRI FLIS, ALBI and major resection	Reported as preoperative; major resection input assumed to be planned	Potential use: planned resection and need for additional FLR assessment	Random internal split (7:3)	AUC 0.746 (development); 0.717 (internal validation)
Fu et al., 2021 (24)	215 HCC patients undergoing major resection; Grade B/C PHLF 23/215	2D-SWE liver stiffness compared with ICG-R15, ALBI, APRI and FIB-4	Preoperative	Potential use: further liver reserve assessment and counseling	Random internal split (150/65; six validation events)	Development AUC 0.795 for liver stiffness; validation specificity 76.3% at 10.35 kPa
Heo et al., 2025 (26)	593 HCC patients with advanced chronic liver disease; Grade B/C PHLF 6.9%	CT-based CSPH substituted into existing EASL and Wang frameworks	Preoperative comparative assessment (CT-CSPH; resection extent treated as planned)	Potential use: further portal hypertension and resection risk assessment	Single-cohort comparative assessment	Wang-model AUC 0.830 to 0.854; EASL AUC 0.694 to 0.724
Chuang et al., 2018 (35)	115 mixed-diagnosis hepatectomies; 90 HCC; study-specific PHLF 13.9% (16/115)	rCER combining parenchymal enhancement with standardized remnant volume; volume calculated using resected specimen weight	After specimen removal (rCER uses resected specimen weight)	Potential use: postoperative risk stratification and monitoring	Assessment in one cohort (no independent validation)	AUC 0.780 for rCER; 0.750 for RLV/SLV (entire cohort; difference not significant)
Theilig et al., 2019 (36)	36 mixed-diagnosis extended right hepatectomies after right PVE; one HCC; any-grade PHLF 14/36	Serial Gd-EOB-DTPA MRI; change in left-lobe relative enhancement from baseline to 14 days after PVE	Preoperative, after post-PVE reassessment	Potential use: reassessment before extended hepatectomy after FLR augmentation	Assessment in one cohort (no independent validation)	AUC 0.854 for any-grade PHLF; 0.880 for Grade C PHLF (entire cohort)
Akabane et al., 2023 (37)	120 patients with marginal ICG-Krem; 95 HCC; any-grade PHLF 24.2% (29/120)	Gadoxetic acid-enhanced MRI rHUI compared with ICG-Krem, ICG-R15, ALBI and ALICE	Preoperative (planned-remnant segmentation)	Potential use: risk stratification in patients with marginal ICG-Krem and reassessment of the resection plan	Five-fold internal cross-validation of the rHUI cutoff (no independent validation)	AUC 0.814 for rHUI (entire cohort); cross-validated sensitivity 68.9% and specificity 88.4% at cutoff 925
Kudo et al., 2025 (38)	333 mixed-diagnosis major hepatectomies; Grade B/C PHLF 6.9% (23/333); PVE subgroup n=114	Gadoxetic acid-enhanced MRI: volume-adjusted increase rate of the remnant liver-to-spleen signal ratio (vaLSRi-rem)	Imaging biomarker: preoperative; adjusted association: end of surgery because operative covariates were included	Potential use: preoperative FLR-function assessment	Assessment in one cohort (no independent validation)	Sensitivity 83% and specificity 80% at vaLSRi-rem cutoff 0.147 (entire cohort); AUC not reported
Li J et al., 2025 (39)	37 mixed-diagnosis minor hepatectomies; 29 HCC; study-specific PHLF 35.1% (13/37)	Whole-liver T1 mapping histogram analysis on Gd-EOB-DTPA MRI compared with ICG-R15 and ALBI	Preoperative	Potential use: further liver reserve assessment	Assessment in one cohort (no independent validation)	AUC in the entire cohort: 0.785 (T1HBP SD); 0.734 (ICG-R15); 0.558 (ALBI score)
Cai et al., 2022 (40)	307 HCC patients from three centers; any-grade ISGLS PHLF 22.5% (69/307)	LPC and LSC from Gd-BOPTA hepatobiliary phase MRI compared with Child-Pugh, MELD, ALBI and sFLR	Preoperative	Potential use: additional functional assessment and resection planning	Multicenter pooled assessment (no validation in a center held out from development)	AUC in the pooled cohort: 0.882 (LPC); 0.782 (LSC); 0.687 (Child-Pugh); 0.729 (MELD); 0.711 (ALBI); 0.747 (sFLR)
Meng et al., 2022 (41)	221 HCC patients from two centers; any-grade ISGLS PHLF 27.1% (60/221)	Biliary REB measured 90 minutes after Gd-BOPTA compared with the parenchymal liver-to-muscle ratio	Preoperative	Potential use: additional functional assessment and resection planning	Multicenter pooled assessment (no validation in a center held out from development)	AUC in the pooled cohort: 0.870 (REB); 0.600 (liver-to-muscle ratio)
Li C et al., 2023 (42)	276 HCC patients; 98.6% Child-Pugh A; any-grade ISGLS PHLF 24%	Radiomics score from whole-liver hepatobiliary phase MRI combined with ALBI and ICG-R15	Preoperative	Potential use: further reserve assessment and planned resection discussion	Random internal split (7:3; 194/82)	Combined-model AUC 0.840 (development); 0.820 (internal validation). Internal-validation AUC 0.710 (clinical model); 0.790 (radiomics model)
Tang et al., 2025 (43)	312 retrospective HCC patients plus 62 prospectively recruited patients	XGBoost selected from 12 algorithms; final model included TBIL, MELD, ICG-R15, platelets, tumor size, portal occlusion time, operation time, LMR, GLR, transfusion, Child-Pugh grade and major hepatectomy; SHAP interpretation	End of surgery (portal occlusion time, operation time and transfusion required)	Potential use: immediate postoperative disposition and monitoring	Randomly selected internal validation subset (30%, n=93; separation from the 312-patient development cohort was not clearly reported), plus small same-center prospective temporal validation	AUC 0.983 (development); 0.981 (internal validation); 0.942 (temporal validation at the same center)
Wang et al., 2022 (44)	875 HCC patients recruited from multiple centers	LightGBM using clinical variables including surgical approach, resection extent and intraoperative blood loss; SHAP interpretation	End of surgery (surgical approach, actual resection extent and blood loss required)	Potential use: immediate postoperative disposition and monitoring	Random internal split (pooled training/validation/test cohorts; 612/88/175)	AUC 0.944 (development); 0.870 (internal validation); 0.822 (test cohort)
Mai et al., 2020 (45)	353 HCC patients undergoing hemihepatectomy; Grade B/C PHLF 24.6%	ANN using platelets, PT, bilirubin, AST and sFLR	Preoperative (planned sFLR)	Potential use: counseling, candidacy and planned FLR assessment	Random internal split (265/88)	AUC 0.880 (development); 0.876 (internal validation)
Xu et al., 2023 (46)	265 hemihepatectomies; mixed diagnoses; any-grade PHLF 34.3%	Multiphase CT convolutional neural network without clinical covariates	Preoperative	Potential use: imaging-based risk assessment before resection	Single-center five-fold cross-validation	AUC 0.793
Zhong et al., 2023 (47)	345 treatment-naive HCC patients; symptomatic PHLF	Clinical-radiomics model using multipatch 2D-SWE features	Preoperative	Potential use: liver reserve assessment before resection	Five-fold cross-validation plus temporal validation at the same center	AUC 0.867 (five-fold cross-validation); 0.822 (temporal validation at the same center)
Jin et al., 2024 (74)	226 hepatectomies; Grade B/C PHLF 10.2%; temporal cohort n=31	Separate ANN models with SHAP: preoperative Child-Pugh grade, bilirubin and creatinine; article-labeled postoperative model additionally used actual resection extent	Preoperative ANN: before surgery; article-labeled postoperative ANN: end of surgery (actual resection extent; no postoperative laboratory input)	Potential use: preoperative model—counseling and baseline risk assessment; update at the end of surgery—postoperative disposition and monitoring	Five-fold cross-validation plus temporal validation in a small cohort at the same center	AUC 0.766 (preoperative, five-fold cross-validation); 0.851 (postoperative, five-fold cross-validation); 0.720 (preoperative, temporal validation at the same center); 0.731 (postoperative, temporal validation at the same center)
Baumgartner et al., 2024 (75)	649 mixed-diagnosis major hepatectomies; Grade B/C PHLF 14.2%	Clinical model using diagnosis, cirrhosis, actual resection type, operation time and transfusion; updated with postoperative-day-1 bilirubin	Clinical model: end of surgery; updated model: postoperative day 1	Potential use: model available at the end of surgery—postoperative disposition and monitoring; POD1 update—serial biochemical surveillance and escalation	Development cohort only (no independent validation)	AUC 0.810 (clinical factors); 0.870 (after POD1 bilirubin)

Clinically relevant PHLF denotes ISGLS Grades B and C unless otherwise stated; any-grade PHLF includes Grades A–C. Validation labels describe the actual study design; pooling data from several centers does not constitute independent geographic external validation. Li J et al. (18), Chuang et al. (35), and Li J et al. (39) used modified or study-specific PHLF endpoints. Chuang et al. calculated remnant volume using resected specimen weight. Theilig et al. included only one patient with HCC. Fu et al. reported the liver-stiffness AUC in the development cohort and cutoff specificity in a small internal validation cohort. Heo et al. evaluated CT-based CSPH within existing prediction frameworks. Jin et al. included only 31 patients in the temporal validation cohort. In Kudo et al., 35 patients developed any-grade PHLF, of whom 23 had Grade B/C PHLF; the adjusted ORs were derived from multivariable analyses that also included operative variables. Time of prediction is determined by the last required predictor. ‘Potential use’ denotes a stage-based interpretation and does not indicate that model-guided management has been shown to improve outcomes.

## 3 Prediction models for selected non-PHLF complications

This section focuses on selected clinically consequential non-PHLF complications for which dedicated prediction models applicable to hepatectomy were identified and could be meaningfully appraised, including bile leakage, massive ascites, intrahepatic or perihepatic abscess, and surgical site infection. It is not intended to provide an exhaustive catalog of all postoperative morbidity. Other minor or transient events and general systemic complications, such as pneumonia, are not discussed in detail because endpoint-specific prediction studies in patients with HCC undergoing hepatectomy remain limited and fragmented. When prediction models are available, they are often derived from general surgical or mixed hepatobiliary populations, use heterogeneous outcome definitions, and lack independent validation in cohorts limited to HCC. The following sections therefore concentrate on complications for which the available evidence permits a clinically meaningful comparison of target populations, predictor timing, validation design, and reported performance.

Broader morbidity models estimate aggregate postoperative risk rather than a specific endpoint. In a retrospective cohort of 245 elective liver resections from one center with mixed indications, Madhavan et al. compared the American College of Surgeons National Surgical Quality Improvement Program (ACS-NSQIP) surgical risk calculator with the Physiological and Operative Severity Score for the enUmeration of Mortality and morbidity (POSSUM). ACS-NSQIP was available before surgery for counseling and planning, whereas POSSUM incorporated operative information and was available only after the procedure for postoperative assessment and resource planning. The reported C-statistics were 0.784 for ACS-NSQIP and 0.624 for POSSUM, although the tools used different definitions of overall morbidity, limiting direct comparison (53). Composite morbidity also combines complications with different mechanisms and cannot replace endpoint-specific assessment of PHLF, bile leakage, ascites, or infection.

### 3.1 Bile leakage

Bile leakage is a common major local complication after hepatectomy. The 2011 ISGLS consensus defined it as drain-fluid bilirubin at least three times the serum concentration on or after postoperative day 3, or the need for radiologic or operative intervention because of a biliary collection or bile peritonitis; severity is graded A–C by its effect on management (54). Clinically relevant Grade B/C leakage varies widely across cohorts, with rates typically around 5–7% in standard hepatectomy populations without biliary reconstruction, and is more frequent after technically high-risk resections or biliary reconstruction (55).

Reported risk factors include impaired liver function, hypoalbuminemia, diabetes, central or extended hepatectomy, biliary-enteric anastomosis, repeat resection, prolonged operative time, and major blood loss. Large-database and meta-analytic evidence indicates that operative anatomy and technique are central determinants of leakage risk (56, 57). These features support selective use of intraoperative leak testing, drainage, and postoperative surveillance in higher-risk patients.

Early clinical risk scores used information available at different perioperative stages. The Patkar four-factor score included operative blood loss and histologic steatosis and therefore was not fully available intraoperatively; it stratified Grade B/C bile leak risk from 2.79% (score 0) to 100% (score 4, based on one patient); a model AUC was not reported (58). A separate four-factor Bile Leak Risk Score (BLRS), which included operative time and was available at the end of surgery, was later developed and validated via temporal validation at the same center (59). Both tools share key limitations: neither reported a full set of discrimination and calibration metrics, and both were developed in cohorts with mixed diagnoses rather than populations limited to HCC.

More recently, machine learning has been applied to larger, multi-institutional datasets to improve performance and generalizability. Altaf et al. trained an XGBoost model on 20,570 NSQIP patients, with independent geographic external validation across 2,253 patients from 19 international centers. The model achieved AUCs of 0.748 (training), 0.719 (test cohort), and 0.711 (independent geographic external validation), paired with SHAP-based interpretability and a publicly available online calculator (60). While the large, geographically diverse sample strengthens the assessment of geographic transportability, the cohort with mixed diagnoses limits direct extrapolation to HCC surgical populations.

Important evidence gaps remain. Most tools were not developed and independently validated specifically in HCC, and the role of background cirrhosis may therefore be incompletely represented. We identified no dedicated, externally validated tool specifically for laparoscopic hepatectomy and no prospective implementation study showing that care guided by a prediction model reduces clinically relevant bile leakage. Future studies should prioritize independent geographic external validation in multicenter HCC cohorts and prespecified links between estimated risk and preventive management.

### 3.2 Postoperative ascites and intra-abdominal infection/abscess

Prediction models for ascites and intra-abdominal infection remain at an earlier stage than those for PHLF or bile leakage. Definitions of “massive ascites” and intra-abdominal infection vary across studies; risk is modified by fluid management, diuretic use, antibiotic prophylaxis, and operative events; and abscess formation may occur downstream of postoperative events such as bile leakage. These features reduce the performance and transportability of purely preoperative models.

In the 232-patient HCC cohort reported by Men et al., massive ascites occurred in 28.4%; a four-variable nomogram incorporating platelet count, aspartate aminotransferase (AST), portal hypertension, and operative time achieved an AUC of 0.837 after bootstrap internal validation, with portal hypertension being the strongest predictor (odds ratio [OR] 5.288) (61). Zhu et al. studied 4,621 patients with HCC across 16 centers; intrahepatic or perihepatic abscess occurred in 3.3% and was associated with 11.7% 90-day mortality. Their six-variable nomogram achieved an apparent C-index of 0.747 and a bootstrap-corrected C-index of 0.731, with diaphragmatic incision the strongest predictor (OR 3.42) (62). Both models included portal hypertension-related information, but neither underwent testing in an independent external cohort or a prospective clinical-utility study.

### 3.3 Surgical site infection

Surgical site infection (SSI) after hepatectomy remains less extensively studied than post-hepatectomy liver failure. The Centers for Disease Control and Prevention (CDC) framework classifies SSI into superficial incisional, deep incisional, and organ/space infection; however, definitions and surveillance windows vary across hepatectomy studies, limiting direct comparability (63). This heterogeneity is particularly relevant in liver surgery, where organ/space SSI may reflect secondary infectious processes rather than primary wound contamination.

Mechanistically, incisional SSI is influenced primarily by patient-related and perioperative factors, whereas organ/space SSI may develop secondary to intra-abdominal complications such as bile leakage, devitalized tissue, or infected collections. This distinction is important because the predictors and timing of incisional and organ/space infection may differ.

Tang et al. developed one of the few SSI prediction tools with independent geographic external validation in patients with HCC, drawing on two geographically distinct institutional cohorts with an overall SSI incidence of approximately 12%. Their nomogram incorporated four independent predictors — operative time, preoperative albumin, repeat hepatectomy, and American Society of Anesthesiologists (ASA) physical status — and achieved C-indices of 0.860 in the derivation cohort and 0.840 in the independent geographic external validation cohort (64). Larger multicenter evidence has emerged more recently. Shen et al. developed an SSI model for HCC across 15 hepatobiliary centers in China, using routinely available preoperative and intraoperative variables. Although the study spanned 15 centers, the model was evaluated only with internal validation; independent testing across different healthcare systems remains needed (65). Preliminary evidence from a multicenter conference abstract by de Klein et al. included 982 liver resections and reported an SSI incidence of 8.1%, encompassing incisional and organ/space infection (66). The authors excluded postoperative bile leakage from the preoperative nomogram. However, the abstract did not report validation or discrimination results, and its applicability to patients with HCC remains uncertain.

To date, no dedicated machine learning model for SSI after hepatectomy with robust external validation has been established. Importantly, several existing models include intraoperative variables such as operative time, which limits their use as purely preoperative decision support tools. Future studies should explicitly differentiate incisional and organ/space SSI, incorporate intermediate postoperative events such as bile leakage, and evaluate whether dynamic or sequential prediction frameworks improve performance over baseline preoperative models.

### 3.4 Comparative maturity of prediction models across endpoints

PHLF is the most comprehensively studied endpoint, with evidence spanning clinical and serum scores, imaging and functional assessment, multivariable models, and machine learning, as well as several externally validated tools and online calculators. Bile leakage has an intermediate evidence base, including clinical risk scores and a machine learning model with independent geographic external validation, but still lacks sufficient validation in HCC cohorts. Postoperative ascites and intra-abdominal infection/abscess remain supported mainly by regression nomograms with internal bootstrap validation and no independent external testing. SSI has one nomogram with independent geographic external validation but no dedicated machine learning model with robust external validation. These differences reflect endpoint standardization, event frequency, predictor availability, and the extent to which risk is driven by measurable patient factors rather than operative technique and postoperative events. The overall morbidity comparison and representative models for selected non-PHLF complications are summarized in **Table 2**.

**Table 2:** Representative prediction models for overall morbidity and selected non-PHLF complications.

Study/outcome	Population and endpoint	Approach and principal inputs	Time of prediction	Intended clinical action	Validation classification	Reported performance
Overall morbidity - Madhavan et al., 2018 (53)	245 elective liver resections; mixed indications; ACS-NSQIP ‘any complication’ 27.3%; POSSUM-defined 30-day morbidity 38.3%	ACS-NSQIP calculator using preoperative patient and planned-procedure variables; POSSUM using physiological and operative-severity variables; both variable sets were also refitted in the same cohort	ACS-NSQIP: preoperative; POSSUM: end of surgery	Potential use: ACS-NSQIP—counseling and operative planning; POSSUM—postoperative management and resource planning	Single-cohort evaluation of the original calculators; same-cohort logistic refitting without independent validation	Original calculators: C-statistic 0.784 (ACS-NSQIP) and 0.624 (POSSUM); same-cohort refitted models: 0.810 and 0.767, respectively
Bile leakage - Patkar et al., 2023 (58)	862 mixed-indication resections; 61 Grade B/C leaks	One-point score: biliary-enteric anastomosis, central/extended hepatectomy, histologic steatosis and blood loss ≥2,000 mL	Postoperative/retrospective (blood loss, actual procedure and histologic steatosis required)	Potential use: risk reassessment after pathology is available; immediate surveillance use remains untested	Development cohort only (no independent validation)	Grade B/C bile leak: 2.79% (score 0) to 100% (score 4; n=1); model AUC not reported
Bile leakage - Riediger et al., 2022 (59)	844-patient development cohort plus same-center later cohort n=142; mixed indications	Age, major resection, FOLFOX/cetuximab pretreatment and operative time	End of surgery (actual resection extent and operation time required)	Potential use: postoperative monitoring and early leak assessment	Temporal validation at the same center	AUC 0.732 (development); 0.755 (temporal validation at the same center); validation sensitivity 68.2% and specificity 75.8%
Bile leakage - Altaf et al., 2025 (60)	20,570 NSQIP cases plus 2,253 external HCC/ICC cases	Preoperative XGBoost; SHAP interpretation and online calculator	Preoperative if procedure fields are entered as planned	Potential use: counseling and planning of perioperative precautions	Independent geographic external validation	AUC 0.748 (training); 0.719 (test cohort); 0.711 (independent geographic external validation)
Massive ascites - Men et al., 2026 (61)	232 HCC patients; massive ascites 28.4%	Platelets, AST, portal hypertension and operative time	End of surgery (operation time required)	Potential use: postoperative fluid-balance and liver function monitoring	Bootstrap internal validation	AUC 0.837
Intrahepatic/perihepatic abscess - Zhu et al., 2025 (62)	4,621 HCC patients from 16 centers; abscess 3.3%	Obesity, diabetes, portal hypertension, major hepatectomy, open surgery and diaphragmatic incision	End of surgery (operative approach, diaphragmatic incision and actual resection extent required)	Potential use: postoperative infection surveillance and diagnostic imaging when clinically indicated	Bootstrap internal validation; no independent external validation	C-index 0.747 apparent; 0.731 bootstrap-adjusted
SSI - Tang et al., 2019 (64)	HCC development n=309; geographic external cohort n=331	Operative time, albumin, repeat hepatectomy and ASA class	End of surgery (operation time required)	Potential use: postoperative SSI surveillance and early diagnostic assessment	Independent geographic external validation	C-index 0.860 (development); 0.840 (independent geographic external validation)
SSI - Shen et al., 2025 (65)	4,124 HCC patients from 15 centers; SSI 9.5%	Obesity, diabetes, portal hypertension, blood loss, open approach, diaphragmatic incision, major resection and operative time	End of surgery (blood loss, operative approach, actual resection extent and operation time required)	Potential use: postoperative SSI surveillance and early diagnostic assessment	Bootstrap internal validation; no independent external validation	C-index 0.733 apparent; 0.725 bootstrap-adjusted
SSI - de Klein et al., 2021 (66)	982 mixed-indication liver resections; SSI 8.1%; conference abstract	Preoperative nomogram: primary liver tumor, planned concomitant ablation or colorectal resection, and abdominal/gastrointestinal comorbidity; bile leakage excluded	Preoperative (planned concomitant procedures)	Potential use: preoperative SSI risk discussion and preventive planning	Conference abstract; validation not reported	SSI 8.1% (80/982); nomogram discrimination and calibration not reported

Riediger et al. used a later cohort from the same center; this was therefore classified as temporal validation at the same center rather than independent geographic external validation. Multicenter model development without a center-held-out test was not considered external validation. The de Klein study was available only as a conference abstract, limiting appraisal of its methods and validation design. Several models include intraoperative variables and are therefore perioperative rather than purely preoperative. Madhavan et al. evaluated overall morbidity using different definitions for ACS-NSQIP and POSSUM; composite morbidity does not replace endpoint-specific prediction. Its refitted models were developed and evaluated in the same cohort and were not independently validated. Time of prediction is determined by the last required predictor. ‘Potential use’ denotes a stage-based interpretation and does not indicate that model-guided management has been shown to improve outcomes.

## 4 Current challenges and future perspectives

Prediction models for post-hepatectomy complications now span simple clinical scores, multimodal nomograms, functional imaging, and explainable machine learning. Nevertheless, model development has progressed unevenly across outcomes, and high apparent discrimination has not consistently translated into externally validated or clinically actionable tools.

### 4.1 Unequal maturity across postoperative outcomes

PHLF is the most extensively studied prediction endpoint because it has a standardized ISGLS definition, strong associations with mortality, and measurable preoperative determinants. Bile leakage has reached an intermediate stage, with clinical risk scores and a large externally validated machine learning model, but prediction remains constrained by its dependence on operative anatomy and technical events. Models for ascites, intra-abdominal abscess, and surgical site infection remain sparse and are predominantly supported by internally validated nomograms. These differences indicate that model performance must be interpreted in the context of endpoint biology, event frequency, and the time at which relevant predictors become available.

### 4.2 External validation, transportability, and recalibration

The most consistent weakness across the literature is limited genuinely independent external validation. Many studies pool patients from several institutions and then perform random train-test splitting, which evaluates reproducibility within the pooled dataset rather than transportability to a new clinical setting; external validation requires evaluation in data not used in model development (67). Independent geographic external validation and appropriately labeled temporal validation are particularly important in HCC because the prevalence of hepatitis B virus (HBV)-related cirrhosis, metabolic dysfunction-associated steatotic liver disease, portal hypertension, and major hepatectomy varies across regions. Future studies should report external discrimination and calibration, assess calibration drift, and provide recalibration strategies before implementation in populations that differ from the development cohort.

### 4.3 Reporting quality and risk of bias: TRIPOD and PROBAST

Methodological rigor should be evaluated alongside model performance. The TRIPOD statement provides a structured framework for transparent reporting of prediction model development and validation, whereas PROBAST evaluates risk of bias and applicability across participant selection, predictors, outcomes, and analysis domains (68, 69). Common concerns in the extracted literature include small event counts, data-driven predictor selection, inadequate handling of missing data, random splitting of modest datasets, incomplete calibration reporting, and the absence of independent validation. Future studies should justify sample size using prediction-model-specific criteria (52), address missingness with principled methods such as multiple imputation when assumptions are defensible (70), preserve continuous predictors rather than selecting data-driven cut points (71), report calibration-in-the-large and calibration slope (72), and make the complete model equation or executable calculator available.

### 4.4 The clinical value of artificial intelligence

Machine learning can model nonlinear relationships and higher-order interactions, but higher AUC values should not be interpreted as proof of superior clinical value. Reported gains are influenced by sample size, outcome prevalence, predictor richness, and validation design. Moreover, explanation methods describe how a model generates predictions but do not establish causal relationships or demonstrate that clinicians can act effectively on those predictions. Artificial intelligence (AI) models should therefore be compared with well-specified regression models using identical predictors and validation datasets, and evaluation should include calibration, net benefit, usability, fairness, and computational requirements rather than discrimination alone.

### 4.5 Dynamic perioperative prediction

Prediction timing determines the clinical question a model can address. Preoperative models use information available before incision and may inform counseling, candidacy, planned resection extent, and additional FLR assessment. Intraoperative or end-of-surgery models add actual resection extent, operative time, blood loss, or transfusion; they can support immediate postoperative disposition and monitoring but cannot guide the initial decision to operate. Early postoperative models incorporate laboratory or physiological responses to resection and update risk for surveillance and escalation. Because predictor availability, intended action, prediction horizon, and sometimes the endpoint differ, AUCs across stages or outcomes should not be used to rank model quality directly. For time-to-event outcomes with repeated predictors, landmarking supports risk updating among patients who remain event-free at successive time points (73).

Initial studies support the feasibility of updating at successive stages, although truly dynamic models remain uncommon. Jin et al. developed separate preoperative and postoperative artificial neural network models for Grade B/C PHLF. Although termed “postoperative” in the original study, the second model incorporated actual resection extent and could be calculated at the end of surgery. Its mean AUC under five-fold cross-validation was 0.851, compared with 0.766 for the preoperative model. However, performance was attenuated in the temporal validation cohort from the same center, with corresponding AUCs of 0.731 and 0.720, indicating limited evidence of transportability (74). In a larger cohort of 649 patients undergoing major hepatectomy, Baumgartner et al. found that adding bilirubin measured on postoperative day 1 to clinical risk factors increased the AUC for Grade B/C PHLF from 0.810 to 0.870 (75). This model provided an early postoperative update but did not constitute continuous dynamic prediction.

Dynamic updating may also be linked to a clinically important endpoint such as 90-day mortality. In a retrospective cohort of 244 patients undergoing hepatectomy for HCC at one center, Lei et al. recorded 13 deaths (5.3%) within 90 days. Child–Pugh score, intraoperative blood loss, peak bilirubin >119 μmol/L on postoperative day 3, and fulfillment of the 50–50 criteria on postoperative day 5 were associated with mortality in multivariable analysis (76). This analysis shows how information available at successive perioperative stages may refine risk assessment, but it was a retrospective risk-factor analysis without model validation rather than a validated dynamic mortality model. The variables measured on postoperative days 3 and 5 cannot inform the initial decision to operate, and the liver dysfunction criteria used in this study should not be treated as interchangeable with ISGLS PHLF grading.

Postoperative phosphate illustrates the difficulty of interpreting a biochemical trajectory in isolation. In a narrative review, Chan et al. found heterogeneous associations between phosphate changes and liver regeneration, PHLF, morbidity, and mortality: some cohorts linked a greater fall in phosphate with recovery, whereas others associated marked hypophosphatemia with adverse outcomes (77). The direction and threshold of these associations varied with measurement time, the inclusion of healthy donors or patients with diseased livers, the extent of resection, and phosphate replacement. Neither lower nor higher phosphate can therefore be regarded as uniformly favorable, and any sequential model would require prespecified sampling times and consideration of supplementation. Broader postoperative changes in carbohydrate, lipid, protein, and energy metabolism may provide candidate signals for future risk updating, but Lee et al. summarized heterogeneous, mainly associative evidence rather than validated serial prediction tools (78). Such trajectories remain a research direction and are not established for routine continuous risk updating.

Taken together, these studies support testing a staged framework that starts with baseline liver function and anticipated future liver remnant, adds actual operative information, and then evaluates selected postoperative laboratory or metabolic measurements. Candidate updates require validation for calibration and net benefit over the preceding stage before they are linked to management. The proposed framework for prediction at successive stages is summarized in **Figure 1**.

**Figure 1:**
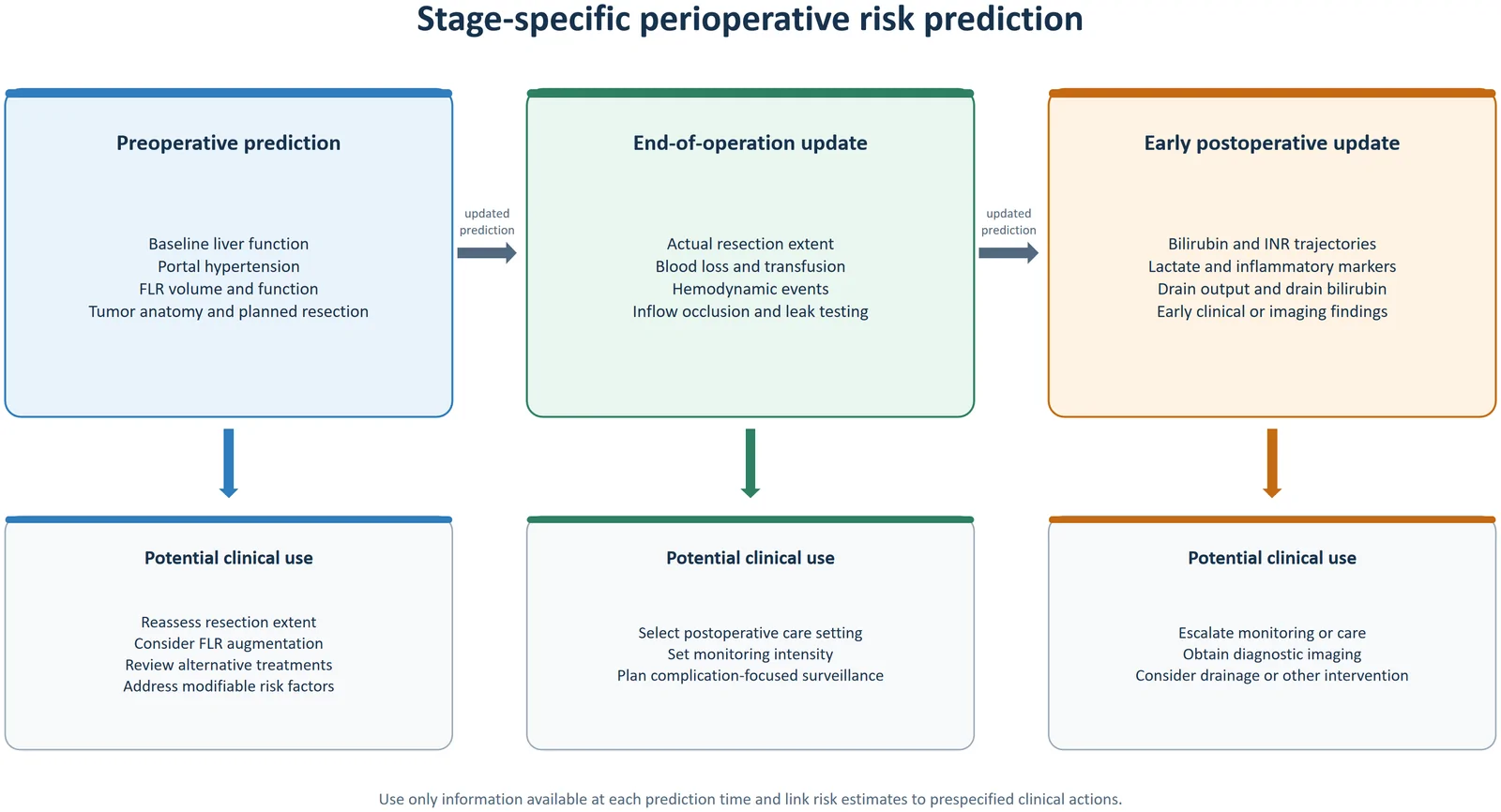
Conceptual framework for stage-specific perioperative risk prediction after hepatectomy. Variables and actions are illustrative; the framework has not been validated as a clinical algorithm.

### 4.6 From AUC to clinical utility

Before surgery, high predicted PHLF risk could prompt reassessment of the planned resection in light of tumor clearance, portal hypertension, and FLR volume and function. Established liver function criteria inform resection extent (5, 14); models may identify patients needing further assessment. Where tumor clearance permits, parenchyma-sparing resection may preserve more remnant tissue (79). If major hepatectomy remains necessary and the FLR is inadequate, PVE may be considered in suitable patients, followed by reassessment of remnant growth and function (6, 36, 38). Associating liver partition and portal vein ligation for staged hepatectomy (ALPPS) is a more intensive option for selected cases; volume gain should not be assumed to equal functional gain (79). A model estimate alone cannot establish which strategy will improve outcomes.

If resection remains unsuitable after this assessment, treatment options should be reviewed according to tumor burden, liver reserve, and patient preferences. Depending on eligibility, these may include thermal ablation for small, suitably located tumors or liver transplantation; other locoregional or systemic treatments require stage-appropriate selection (2). A PHLF model estimates risk after resection and does not directly compare the benefits and harms of these alternatives. For patients proceeding to surgery, risk assessment can also draw attention to potentially modifiable problems, including malnutrition, poorly controlled diabetes, and active chronic hepatitis, for nutritional support and appropriate medical management (6).

Perioperative interventions can alter complication risk independently of risk prediction. In a meta-analysis of eight randomized trials including 590 liver-resection patients with mixed indications, Hai et al. reported fewer overall complications with steroids (OR 0.58, 95% CI 0.35–0.97), but the pooled analyses did not demonstrate a reduction in reported liver dysfunction/failure or hepatobiliary complications (80). A mortality effect could not be estimated because no deaths occurred in the trials reporting mortality. Steroid regimens and endpoints varied, and treatment was not assigned according to a prediction model; these findings do not support routine steroids for patients classified as high risk by a model.

After surgery, an elevated or rising risk estimate could support closer clinical review and serial assessment of bilirubin, INR, and organ function. Early postoperative prediction studies provide a rationale for identifying vulnerable patients before the formal PHLF assessment on or after postoperative day 5 (3, 75). Escalation to a higher level of care should depend on the observed clinical course, operative events, and organ dysfunction as well as the estimated risk. Current evidence does not establish a universal model threshold for intensive care admission or a particular monitoring schedule.

The ultimate purpose of prediction is to improve decisions and patient outcomes, not merely to maximize statistical accuracy. Future research should define explicit actions for each risk category and test pathways guided by model estimates in prospective implementation studies. Decision curve analysis estimates clinical net benefit across threshold probabilities (81), but it cannot replace evidence that a model changes management safely and cost-effectively. Pragmatic trials should evaluate whether care guided by model estimates reduces clinically relevant PHLF, bile leakage, infection, resource use, or mortality. Until such evidence is available, even externally validated models should be viewed as decision support tools rather than autonomous decision makers.

## 5 Conclusion

Risk prediction after hepatectomy for HCC now incorporates clinical and serum scores, imaging and functional assessment, multimodal nomograms, and explainable machine learning. PHLF remains the most extensively studied outcome, whereas models for selected non-PHLF outcomes, including bile leakage, postoperative ascites and intra-abdominal infection/abscess, SSI, and other individual complications, are less mature. Future models should integrate clinical variables, functional imaging biomarkers, and artificial intelligence methods while maintaining interpretability and external validity. Their clinical value should be assessed through calibration, net benefit analysis, and prospective evaluation of clearly defined management pathways rather than marginal gains in AUC alone.
